# Supplementary material for: A comprehensive two-hybrid analysis to explore the Legionella pneumophila effector–effector interactome
Source: mSystems. 2024 Nov 11;9(12):e01004-24. doi: 10.1128/msystems.01004-24 (PMC11651115; doi:10.1128/msystems.01004-24)
Supplement: Fig. S1 — iBFG-Y2H barcode representation and correlation of fusion barcode tags. [file msystems.01004-24-s0001.pdf]

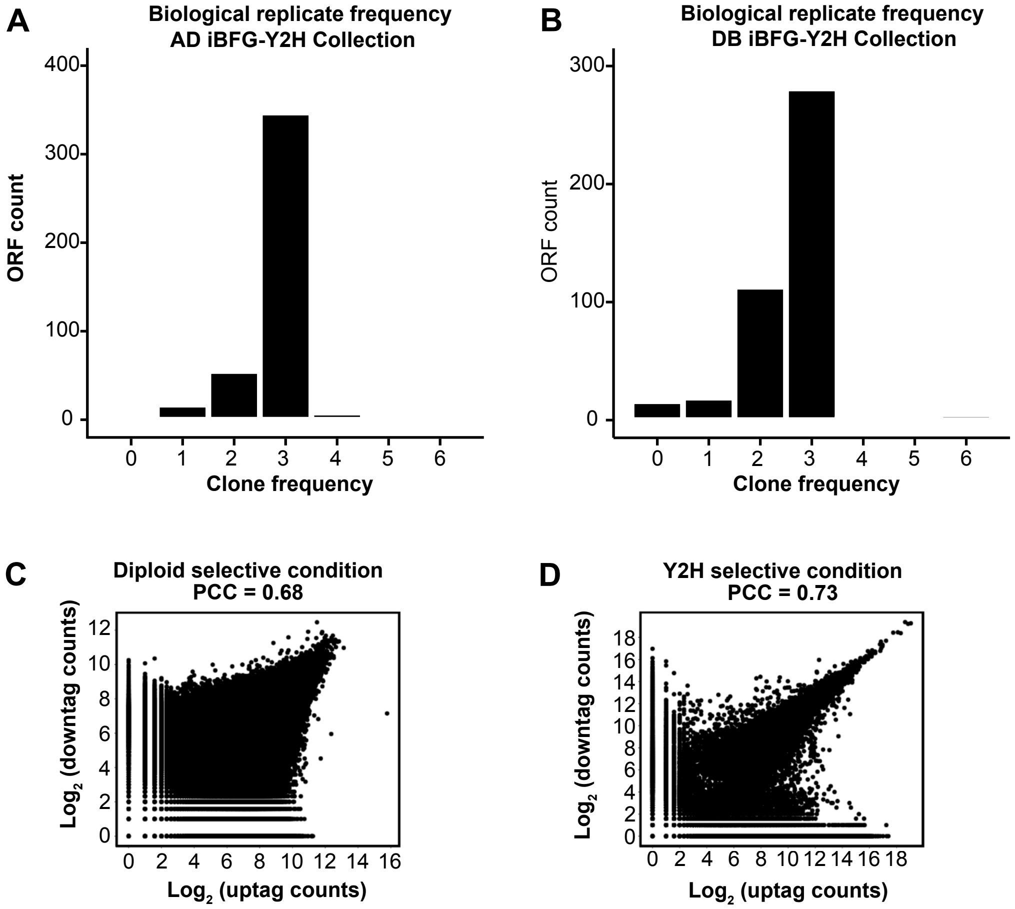

Figure S1: iBFG-Y2H barcode representation and correlation of fusion barcode tags
A, B) The bar graphs show the distribution of barcode representation of ORFs in the AD (**A**) and DB (**B**) collection. The majority of ORFs are represented by at least three uniquely barcoded vectors. **C, D**) Barcode recombination leads to an equal number of up and down fusion barcodes for each unique pair. The scatter plots show the counts of the uptag fusion barcodes plotted against the downtag fusion barcodes and the Pearson correlation (PCC) of the barcode pair abundance across the entire population in the control (**C**) and Y2H selective (**D**) conditions. The uptag and downtag fusions show good concordance, which indicates that there are no major barcode-specific effects due to PCR or sequencing.
