# Supplementary material for: A comprehensive two-hybrid analysis to explore the Legionella pneumophila effector–effector interactome
Source: mSystems. 2024 Nov 11;9(12):e01004-24. doi: 10.1128/msystems.01004-24 (PMC11651115; doi:10.1128/msystems.01004-24)
Supplement: Fig. S2 — Retest of iBFG-Y2H interactions with L. pneumophila effectors. [file msystems.01004-24-s0002.pdf]

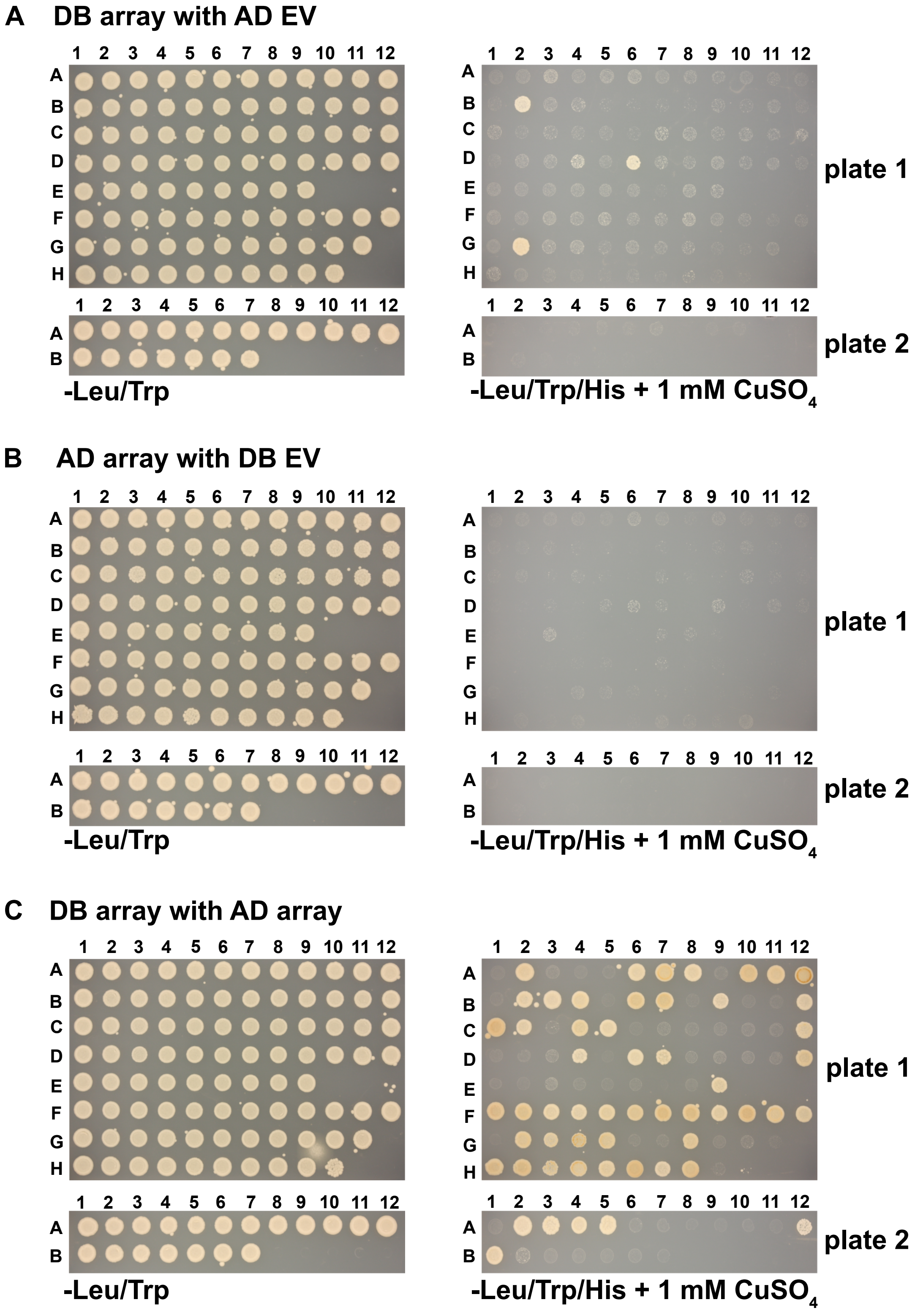

Figure S2: Retest of iBFG-Y2H interactions with *L. pneumophila* effectors
All 107 interaction pairs above the optimal MCC interaction score threshold involving effectors (effector-effector, effector-human, effector-Dot/Icm) and Dot/Icm-Dot/Icm pairs were retested. The vectors were recloned from confirmed Gateway entry clones, transformed to BFG-Y2H strains RY1010 and RY1030 and arrayed in an AD and DB array. A1 in plate 1 and 2 are empty vector controls, see Table S4 for the ORF identities in each spot. **A**) The DB array was mated with AD-EV, spotted on diploid selective (-Leu/Trp medium) and Y2H selective conditions (-Leu/Trp/His + 1 mM $CuSO_4$) and grown for 3 days before imaging. Two effector fusions (DB-Lpg2860 and DB-Lpg2300/LegA3) and one Dot/Icm fusion (DB-IcmS) are autoactivators and can grow on Y2H selective conditions in the presence of the AD empty vector. **B**) The AD array was mated with DB-EV and grown as above. **C**) The DB array was mated with the AD-array and grown as above. Of the 107 interaction pairs, 56 interactions verify in the retest experiment.
